# EEG-based spatio-temporal relation signatures for the diagnosis of depression and schizophrenia

**DOI:** 10.1038/s41598-023-28009-0

**Published:** 2023-01-14

**Authors:** Oded Shor, Amit Yaniv-Rosenfeld, Avi Valevski, Abraham Weizman, Andrei Khrennikov, Felix Benninger

**Affiliations:** 1 Felsenstein Medical Research Centre, Petach Tikva, Israel; 2 Department of Neurology, Rabin Medical Centre, Petach Tikva, Israel; 3 Geha Mental Health Centre, Petach Tikva, Israel; 4 Faculty of Technology, Department of Mathematics, Linnaeus University, Vaxjö, Sweden; 5 Sackler Faculty of Medicine, Tel Aviv University, Tel Aviv, Israel; 6 Present Address: Shalvata Mental Health Centre, Hod Hasharon, Israel

**Keywords:** Diagnostic markers, Applied mathematics, Physics, Biological physics, Depression, Schizophrenia, Neurophysiology, Biomarkers, Diagnostic markers, Schizophrenia, Depression

## Abstract

The diagnosis of psychiatric disorders is currently based on a clinical and psychiatric examination (intake). Ancillary tests are used minimally or only to exclude other disorders. Here, we demonstrate a novel mathematical approach based on the field of p-adic numbers and using electroencephalograms (EEGs) to identify and differentiate patients with schizophrenia and depression from healthy controls. This novel approach examines spatio-temporal relations of single EEG electrode signals and characterizes the topological structure of these relations in the individual patient. Our results indicate that the relational topological structures, characterized by either the personal universal dendrographic hologram (DH) signature (PUDHS) or personal block DH signature (PBDHS), form a unique range for each group of patients, with impressive correspondence to the clinical condition. This newly developed approach results in an individual patient signature calculated from the spatio-temporal relations of EEG electrodes signals and might help the clinician with a new objective tool for the diagnosis of a multitude of psychiatric disorders.

## Introduction

Mental disorders are typically diagnosed based on psychiatric interviews with the patients and their families and on the patients’ documented medical reports, including neurological examinations^1–3^. Since the diagnosis is based to a large extent on subjective assessments and possibly thus delaying diagnosis, therapeutic interventions may be inappropriate or ineffective emphasizing the need for an objective biomarker to establish a firm diagnosis in an early stage of the disease and to help the development of precision and biological psychiatry^4,5^.

Electroencephalography (EEG) is a widely used, inexpensive and well-established technology for assessing brain electrophysiology; it is mainly used in the diagnosis of epilepsy^6,7^. EEG signature activity such as resting-state power, spectral and functional connectivity analyses as well as microstate analyses have been suggested as possibly relevant in diagnosing schizophrenia^8–11^ and major depression^12–16^; however, it is not used in clinical practice for such purposes as of yet.

We suggest using EEG for diagnosing mental disorders. Our approach follows recent developments in dendrographic hologram (DH) theory^17–20^, which is based on representing systems (physical, biological, cognitive) by events generated during discrete periods.

In physics, this approach corresponds to the event-picturing of the universe^21–23^. Events are outcomes or patterns of outcomes of measurements. Bohr repeatedly highlighted the role of the phenomenon—the event of the individual outcome of a measurement, for instance, a dot on the photo-emulsion screen in an interference experiment with photons or electrons^24^. DH theory is heavily based on the methodology of quantum theory; however, the quantum microsystems cannot be used directly. Thus, the outcomes of measurements are obtained in a process of complex interactions among systems and measurement devices, for example, interactions between photons and photodetectors^25^.

To obtain knowledge on a patient’s mental state, the neurologist or psychiatrist uses various observation techniques like EEG or MRI, or asks questions, which in this case are considered measurements. In cognitive studies, outcomes of mental observations are not intrinsic properties of the human psyche, but rather events (phenomena, according to Bohr) associated with brain function^26^. It thus seems reasonable to use the event approach from physics for the study of system behaviour, cognition and the human psyche.

We continue Bohr’s work involving a modern interpretation of quantum mechanics for obtaining information. According to Bohr, the outcomes of measurements are not the objective properties of systems. They quantitatively represent interrelations between a system and an observer (measurement device of the observer, e.g., EEG device). The knowledge (or information) obtainable on a system by the observer (may it be quantum, classical or biological) is extracted from experiments (collection of data, information). Thus an EEG device will interrelate with a patient and obtain knowledge from him in the same manner as subjective assessments based on a structured interviews in psychiatry. We adhere also to Wheeler’s work^27^ with his “it from bit” program of reconstruction of physics and the methodology of information biology, which started with Johnson’s^28^ characterization of information theory as a general calculus for biology. According to Gatenby and Frieden, life without matter and energy is impossible^29^. Johnson claimed that life without information is also impossible^28^. DH theory presents a novel application of the aforementioned ideas by using hierarchic clustering algorithms to represent events as trees—dendrograms^17,18^. For concrete experimental data, trees are finite, but as shown by Shor et al., in theory, infinite trees exist as well^17–20^. For practical applications, the most useful trees are homogeneous ones, where the number of edges for each vertex is identical. A p-adic tree structure is characterized by one incoming edge and p outgoing edges for each vertex, where p is a natural number larger than one (p > 1). p-adic trees can be imbued with an algebraic structure, including addition, subtraction, multiplication and, for prime p’s, even division. The simplest trees are 2-adic ones. We used clustering algorithms to generate such trees. The trees can be invested with ultrametric topology, whose distinguishing property is that any two balls are either disjointed or one is a sub-ball of the other.

Furthermore, p-adic trees imbued with algebra are known as rings of p-adic numbers^30^. They have been widely used in areas of physics such as string theory, cosmology, general relativity and quantum theory^31–37^. Parisi used p-adic numbers in the mathematical formulation of the replica symmetry method that serves as the basis of the theory of complex disordered systems^38–41^. p-adic trees were also used in biology^42–48^, for instance, to model information-processing in the brain and conceptualizing human memory retrieval^42^. Moreover, Freud's idea on the splitting of cognitive processes into two closely connected domains (consciousness and subconsciousness) is modelled based on the p-adic field and shows the process of thinking as a random dynamical process^46^.

The transition from theoretical modelling to practical applications is presented in an article by Shor et al.^49^. Here, the clustering algorithms and generated dendrograms thereof were used to represent hierarchic relations between events that consist of EEG measurements. The novel technique is based on a time series of dendrograms instead of straightforward use of an EEG-output time series. The medical diagnostic algorithm was based on a relatively rough dendrogram analysis known as quantum potential, which is a central concept of Bohmian mechanics. We interpreted the data according to Bohm and Hiley^26^. It should be noted that quantum probability and information are widely used in the modelling of cognition, decision-making, psychology and social sciences^42,50–53^ and are known as quantum-like to distinguish them from genuine quantum theory of cognition^54,55^. Bohr emphasized the possibility to apply quantum methodology in biology^56^ and Shor et al. described the quantum-like model as a dendrogramic configuration space^20^.

We define the fundamental concept of DH theory as the “event”. An event can be any single measurable feature of a sample space or a number of such features. Each event is related to every other measurable event in the specific sample space. For example, the calcium level in a specific blood test is related to the calcium level in a previous blood test.

Using objective tools to classify psychiatric patients is a great challenge. We here present a novel algorithm using EEG to differentiate patients suffering from psychiatric disorders (depression and schizophrenia) and control participants. By means of a distance metric and a linkage algorithm as described in detail in the methods section, relations between events can be represented as tree structures called dendrograms.

We show that patients’ personal DH signatures based on EEG recordings accurately differentiate patients with diverse psychiatric disorders. This personal DH signature provides an accurate picture of hierarchic interrelations between events generated by patients’ brains, leading to more precise diagnosis of psychiatric disorders.

## Methods

The study adhered to the rules and regulations of the Helsinki Declaration and was approved by the Institutional Review Board (IRB) of the Rabin Medical Centre, Petach Tikva, Israel (0275-20-RMC). The study was approved as retrospective clinical, and the need for informed consent was waived by the ethics committee. All patient data were fully anonymized before review.

### Participant groups

Electronic medical health records (EMHR) were used to identify all participants that underwent at least one EEG examination between the years 2011 and 2019. The participants were then divided into the following groups: control participants undergoing EEG due to indications unrelated to neuropsychiatric diseases, participants with a diagnosis of major depressive disorder (MDD) and patients diagnosed with schizophrenia. A total of 166 participants (average age: 52.4 ± 18.7 years; range: 18–91 years; 98 (59.4%) female) were included in the study:

1. Controls: Participants (n = 96; age: 52.2 ± 16.8 years; range: 19–80 years; 63 females) undergoing EEG due to indications unrelated to neuropsychiatric diseases. Exclusion criteria for this group included diagnosis of depression or schizophrenia, bipolar disorder, substance abuse, psychiatric or general medical conditions requiring hospitalization, history of epilepsy or conditions requiring anticonvulsants, ECT, vagal nerve stimulation, or transcranial magnetic stimulation (TMS), history of traumatic brain injury and history or imaging findings of cerebrovascular diseases including ischaemic and haemorrhagic stroke.
2. Depression: Participants with a diagnosis of major depressive disorder (MDD) hospitalized during the index time. This diagnosis had been established by two senior psychiatrists according to DSM-IV and DSM-V criteria following a psychiatric interview where the severity of depression was found to be at least moderate. In addition, the participants (n = 28; age: 69.7 ± 14.8 years; range: 33–91 years; 20 females) had to have had at least 1 previous major depressive episode, prior to age 30—namely, the index episode was a recurrent one.
3. Schizophrenia: Diagnosis of schizophrenia had been established by two senior psychiatrists according to the ICD-10 criteria. In addition, the participants (n = 42; age: 41.4 ± 16.8 years; range: 18–76 years; 15 females) had to be hospitalized during the index time.

### EEG data acquisition

The EEG recordings were retrieved from the EMHR of all patients. EEGs had been recorded in a routine clinical setting by an experienced EEG technician. All the patients included in the study had undergone EEGs between 8 am and 1 pm using a Nihon Kohden surface EEG (19-electrode standard according to the international 10–20 electrode placement system) with a sampling frequency of 500 Hz (Nihon Kohden, Japan). During the EEGs, patients had been awake in a resting position with open or closed eyes.

To extract the hierarchical relational dendrogram from the patients’ EEG signals, we converted the raw EEG data from the 19 active electrodes $(\mathrm{elec})$ into the European Data Format (EDF). Data then was filtered to remove the 50-Hz mains signal and further filtered with a high-pass filter of 1 Hz. Data was used without removing muscle artefacts or clarifying open or closed eyes state. To assess the possibility that artefacts in the EEG recordings might account for group differences, we used artefact removal algorithm as described below. For removal of independent components (ICs) of artefacts related to ocular, muscular, cardiac activities, or other movement artifacts, we used EEGLAB toolbox^57^ and its *FASTER* plugin^58^. In short, the pre-processing procedure involved filtering all recordings by a high-pass filter of 1 Hz as well as a notch filter of 50 Hz. This was followed by noisy channels and independent components (ICs) of artifacts related to ocular movements, muscle artifacts, cardiac activities or other movement artefacts, identification by the Faster algorithm. The identified noisy channels were interpolated and artefactual ICs were removed, respectively.Analysis was done using MATLAB software (Mathworks, Natick, MA).

### Receiver operating characteristic (ROC)

ROC analysis is used clinically to quantify how accurately medical diagnostic tests (or systems) can discriminate between two states. The ROC curve shows the trade off between the true positive fraction (TPF) and false positive fraction (FPF) as one change the criterion for positivity. The area under the curve (AUC) summarizes the entire location of the ROC curve Thus the AUC is a measure of sensitivity, specificity and validity of diagnostic tests. Roc analysis was accomplished with MATLAB software (Mathworks, Natick, MA).

### Universal dendrogram analysis and calculation of patients’ personal universal DH signatures (PUDHSs)

**Figure 1:**
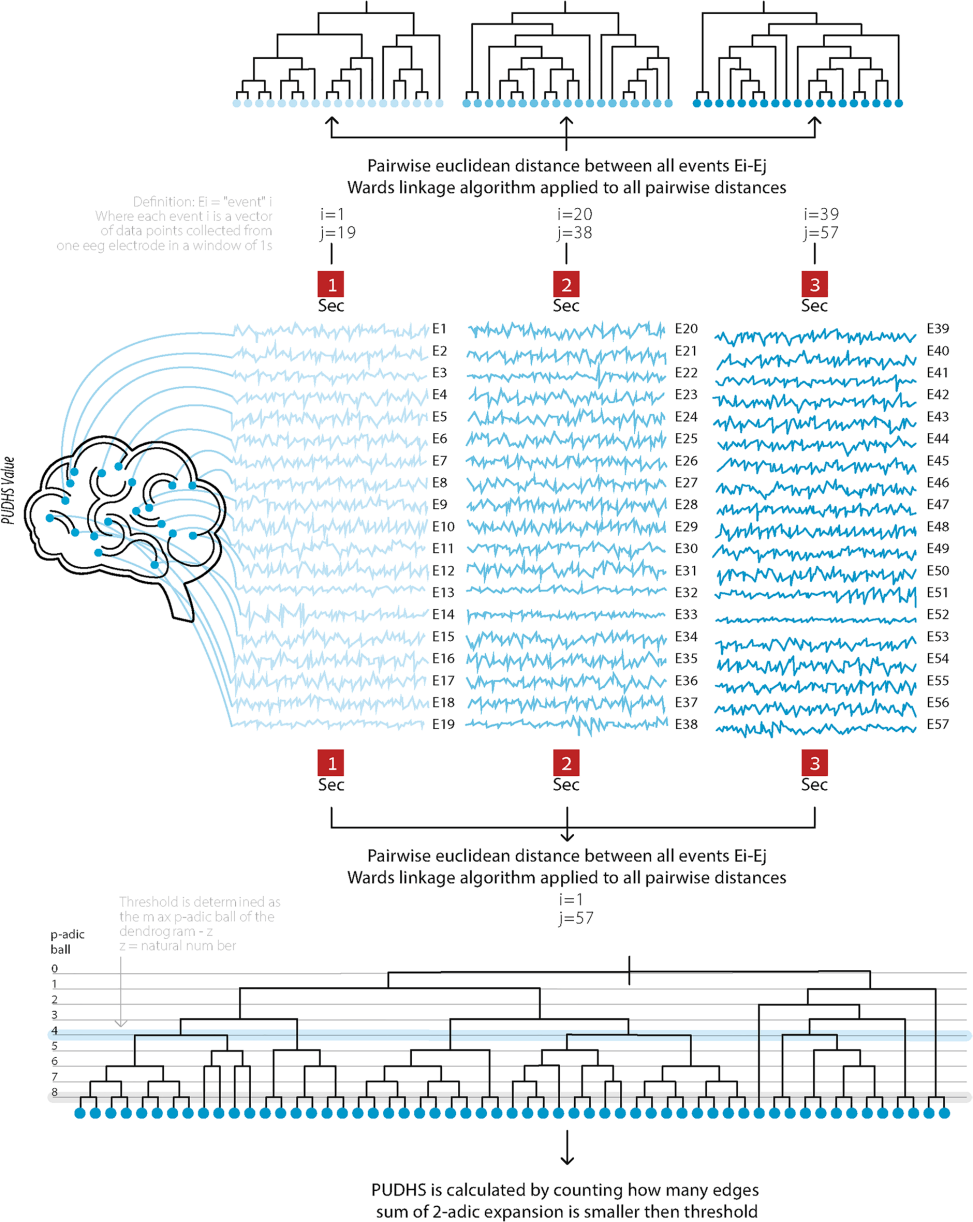
From EEG signal via dendrograms to PUDHS values—an illustration. A simple example showing a time series of 3 dendrograms (up) from three 1 s widows. Each dendrogram has 19 branches corresponding to the 19 electrodes. lower dendrogram was constructed by pairwise distances of 57 segments of EEG data meaning (3 windows × 19 segments in each window). PUDHS values are calculated by counting how many branches sum of 2-adic expansion is lower than threshold (blue thick horizontal line at the bottom dendrogram).

For each electrode in a person’s EEG (19 in total), we chose a 1 s window consisting of 500 data points (EEG sampling rate of 500 Hz). Each person’s universal dendrogram was constructed by calculating the pairwise Euclidean norm distances between all 1-s windows across all 19 electrodes (see an illustration in Fig. 1). Then a Ward linkage algorithm was employed, resulting in a single personal universal dendrogram ${(\mathrm{D}}_{\mathrm{universal}}$). For each branch of each ${\mathrm{D}}_{\mathrm{universal}}$ we calculated the sum of its p-adic expansion:

1 $${\mathrm{V}}_{\mathrm{i}}=\sum_{\mathrm{j}=0}^{\mathrm{k}}{\mathrm{a}}_{\mathrm{j}}\times {2}^{\mathrm{j}} \quad \mathrm{ where }\,\, {\mathrm{a}}_{\mathrm{j}}=\mathrm{1,0}$$

Thus, ${\mathrm{V}}_{\mathrm{i}}$ is a natural number that represents the relation between ${\mathrm{event}}_{\mathrm{i}}\mathrm{ and \, all \,  even}{\mathrm{t}}_{\mathrm{j}}\mathrm{s  \, where \,  j}\ne \mathrm{i}$.

Consider a dendrogram represented by a (finite) 2-adic tree: the root of the vertex indicates the origin of the 2-adic coordinate system, and each node has one edge going into it and two edges coming out of it. Branches are pathways of combined edges that go from the root edge to an edge at the bottom level of the tree. Each branch is labeled by a binary string of 0 s and 1 s, composing a natural number that encodes the branch (or a point at its bottom level). The set of all branches in the tree is a two-dimensional structure called the dendrogram topology. One concrete system of branch-labeling known as the prefix-code, widely used in information theory, is also used here. The 2-adic metric is the distance between natural numbers, which is determined by the hierarchic structure of the tree. The distance between two branches is determined by their common root-branch: the longer the common root-branch, the shorter the distance.

This 2-adic representation uniquely determines a specific branch, say branch i or event i, as well as the branch’s/event’s relations to all other branches/events. We label this 2-adic number for branch i/event i as Vi.

It should be kept in mind that we currently analyze the relations between EEG-outputs (events) rather than the absolute magnitude of the outputs The relations are expressed by 2-adic numbers, each representing a complex context of spatial (locations of 19 electrodes) and temporal dynamics of the state of the brain.

Each patient’s EEG recording is represented as a hierarchic relational dendogram. A time window of 1 s was chosen for all analyses. According to the frequency of a sample of each patient’s EEG recording, for each of the patient’s 19 electrodes, we prepared 500 vectors of size 1 s × (frequency of sampling), resulting in 500 s of recording divided into 19 × 500 vectors. Thus, a matrix of 9500 row vectors was obtained, with each vector representing an “event” whose relations to all other vectors were yet unknown. To reveal these relations, first pairwise Euclidean distance between all events (9500 × 9499/2 such distances) was calculated. Then the events were linked hierarchically according to their Euclidean distance, resulting in a dendrogramic tree, each branch of which represents an event (a vector of size 1 s × (frequency of sampling)).

As noted above, each branch can be represented by a 2-adic expansion that encodes its relations to all other branches (events) and the sum of an event’s 2-adic expansions is a natural number. Thus, 9500 such natural numbers, fully representing the dendrogram topology, were obtained for each patient, defining the personal universal dendrogram.

To evaluate the topological structure of each patient’s personal dendrogram, a threshold number must be defined. The threshold number dissects the universal dendrogram into subsystems as follows (further discussed in the results section):

2 $${\mathrm{T}}_{\mathrm{personal\, universal\, dendrogram}}={2}^{\mathrm{maximal\,}2-\mathrm{adic\, ball\, of\, personal \,dendrogram}-\mathrm{z}}$$

Here, $\mathrm{z}$ is a natural number (a pre-chosen free parameter of the model) set to the same value for all patients, where personal universal dendrogram is a single dendrogram constructed by all events recorded from a patient. Moreover, 2-adic ball of dendrogram branch Vi is defined by ${\mathrm{log}}_{2}\lfloor \left(Vi \,of\, dendrogram \, branch\right)\rfloor$ where

3 $${\mathrm{maximal\,}2\mathrm{-adic \, ball \,of \,the \,dendrogram}=\lfloor \mathrm{log}}_{2}(\mathrm{max \,Vi \,of \,dendrogram})\rfloor $$

In this approach, ${\mathrm{T}}_{\mathrm{personal\, universal\, dendrogram}}$ is determined individually for each patient’s dendrogram and varies from patient to patient. Once the threshold was defined, we recorded for each patient the number of branches that were smaller than the threshold.

The patient’s personal universal DH signature (PUDHS) is given by the quantity:

4 $$\mathrm{PUDHS}=\mathrm{number\, of \,edges}, {\mathrm{V}}_{\mathrm{i}} < {\mathrm{T}}_{\mathrm{personal\, universal\, dendrogram}}$$

To calculate $\mathrm{PUDHS}$, we chose a natural number between 1 and 8 as the value of each patient’s parameter $\mathrm{z}$.

### Block dendrogram analysis and calculation of patient’s personal block DH signatures (PBDHSs)

As the resources (both computational and time) for constructing a large universal dendrogram become the limiting factor for dendrograms with more than 100 000 branches, we created a block dendrogram DH signature that is much more efficient: we decomposed the previously described dendrogram into a time series of blocks of dendrograms. Following, the corresponding number of edges created by 19 electrodes (9500) was reduced significantly according to the block size: for each of the 19 electrodes, we chose a window of 1 s which consisted of 500 data points. Furthermore, we chose how many windows (which are measured in seconds) would be included in the dendrogram (1, 3, 5 or 10). We then created a dendrogram with the corresponding number of edges to these 19, 57, 95 or 190 (see an illustration for 1 and 3 windows in Fig. 1). Each such dendrogram is constructed by pairwise Euclidean norm distances between all the 1-s windows of the 19 electrodes. Then, Ward’s linkage algorithm is employed with a single resulting dendrogram with 19, 57, 95 or 190 branches. Using this procedure, we analyzed for each patient 500 s in the block dendrograms of each of the various sizes. The total amounts to a time series of 500, 167, 100 or 50 dendrograms. For a particular size of dendrogram blocks, we obtained the patient’s personal block DH signature, which is defined as:

${\mathrm{D}}_{\mathrm{i}} , \quad \mathrm{ where\,  i}=\mathrm{1,2}..\mathrm{n}$ and n = number of block dendrograms in the dendrogramic time series (in our calculations, n = 500, 167, 100, 50).

Each dendrogram consists of:

$$\mathrm{j}=\mathrm{1,2}..\mathrm{m \,number\, of \,edges }(\mathrm{in\, our\, calculations},\mathrm{ m}=19, 57, 95, 190)$$

For each branch, we calculate the sum of its p-adic expansion:

$${\mathrm{V}}_{\mathrm{i}}=\sum_{\mathrm{j}=0}^{\mathrm{k}}{\mathrm{a}}_{\mathrm{j}}\times {2}^{\mathrm{j}} \quad \mathrm{ where }\,{\mathrm{a}}_{\mathrm{j}}=\mathrm{1,0}$$

Thus, ${\mathrm{V}}_{\mathrm{i}}$ is a natural number that uniquely represents the relation of event_i_ to all other event_j_s.

Following, n vectors of ${\mathrm{natural\, numbers \,V}}_{i}$ each of size m that represent the topology of each dendrogram ${\mathrm{D}}_{\mathrm{i}}$.

For each of the vectors, we set 2 thresholds: ${\mathrm{T}}_{\mathrm{dendrogram}}$ (intra-system threshold) and ${\mathrm{T}1}_{\mathrm{dendrogram}}$ (inter-system threshold), defined as follows.

5 $${\mathrm{T}}_{\mathrm{dendrogram}}={2}^{\mathrm{maximal\,}2-\mathrm{adic\, ball \,of \,a \,particular\, block \,dendrogram \,in \,time \,series}-\mathrm{z}1}$$

where the free model parameter $\mathrm{z}1$ was a specific natural number that was identical for all patients. z1 set the 2-adic ball level lower than that of the dendrogram ball.

The threshold ${\mathrm{T}}_{\mathrm{dendrogram}}$ was determined individually for each patient and for each patient’s dendrograms (relational block subsystem).

The 2-adic ball value of each dendrogram was calculated as: $\lfloor ({\mathrm{log}}_{2}(\mathrm{max\, Vi\, of\, dendrogram}))\rfloor$

6 $${\mathrm{T}1}_{\mathrm{dendrogram}}={2}^{\mathrm{maximal\,}2-\mathrm{adic \,ball\, of\, all\, block \,dendrograms \,in \,time \,series}-\mathrm{z}2}$$

where the model parameter $\mathrm{z}2$ was a natural number that was identical for all patients.

z2 set the 2-adic ball level lower than that of the maximal ball of all block dendrograms.

The threshold ${\mathrm{T}1}_{\mathrm{dendrogram}}$ was determined for each individual patient and varied among patients according to the parameters of each patient’s relational block subsystem.

The maximal p-adic ball value of all dendrograms was calculated as

$$\lfloor ({\mathrm{log}}_{2}(\mathrm{max \,Vi\, from \,all\, dendrograms\, in\, the\, patient\, time \,series}))\rfloor $$

and the ${\mathrm{T}1}_{\mathrm{dendrogram}}$ was set accordingly for all dendrograms.

Each dendrogram in the dendrogramic time series was marked by two numbers, E and E1, with values in the range 1–19 × number of windows. E indicated how many branches were smaller than the inter-system threshold—${\mathrm{T}}_{\mathrm{dendrogram}}$, while E1 indicated how many branches were smaller than the intra-system threshold—${\mathrm{T}1}_{\mathrm{dendrogram}}$.

For each value n in the range 1–19 × number of windows, we constructed a histogram F with bins centred at 1,2…n. Each bin included a number that indicated the number of systems dendrograms in the time series whose E value was identical to the value of that bin’s centre-point. A similar histogram, F1, was constructed for the E1 values. For histogram F, we chose randomly a fixed number, h, of bin centres. The chosen bin centres values were kept in the vector p with length h. For histogram F1, we again randomly chose the same fixed number, h, of bin centres. The chosen bin centres values were kept in the vector p1 with length h.

The patient’s personal block DH signature (PBDHS) was then calculated as follows:

7 $$\mathrm{PBDHS }=\mathrm{max}((\mathrm{F}\left(\mathrm{p}\right)+1)*(\mathrm{F}1\left(\mathrm{p}1\right)+1))/\mathrm{ mean}((\mathrm{F}\left(\mathrm{p}\right)+1)*(\mathrm{F}1\left(\mathrm{p}1\right)+1))$$

This formulation makes it possible to combine the $\mathrm{PBDHS}$ and the two thresholds (inter- and intra-system), each of which reveals various features in different p-adic order levels of the block dendrograms, into one signature score.

To maximize the discrimination levels between groups, we calculated the PBDHS with various z1 and z2 combinations. For each combination, we randomly chose different p and p1 bins out of the full histograms F and F1 and repeated these 1 million times. We thus obtained 64 × 3 million (3 different pairs of groups), and ROC AUC values for the differences between pairs of patient groups were calculated using PBDHS values. The sum of AUC values of the 3 groups was calculated for each run.

### Ethical publication statement

We confirm that we have read the Journal’s position on issues involved in ethical publication and affirm that this report is consistent with those guidelines.

## Results

### Characterization and differentiating of participant groups according to *PUDHS*

**Figure 2:**
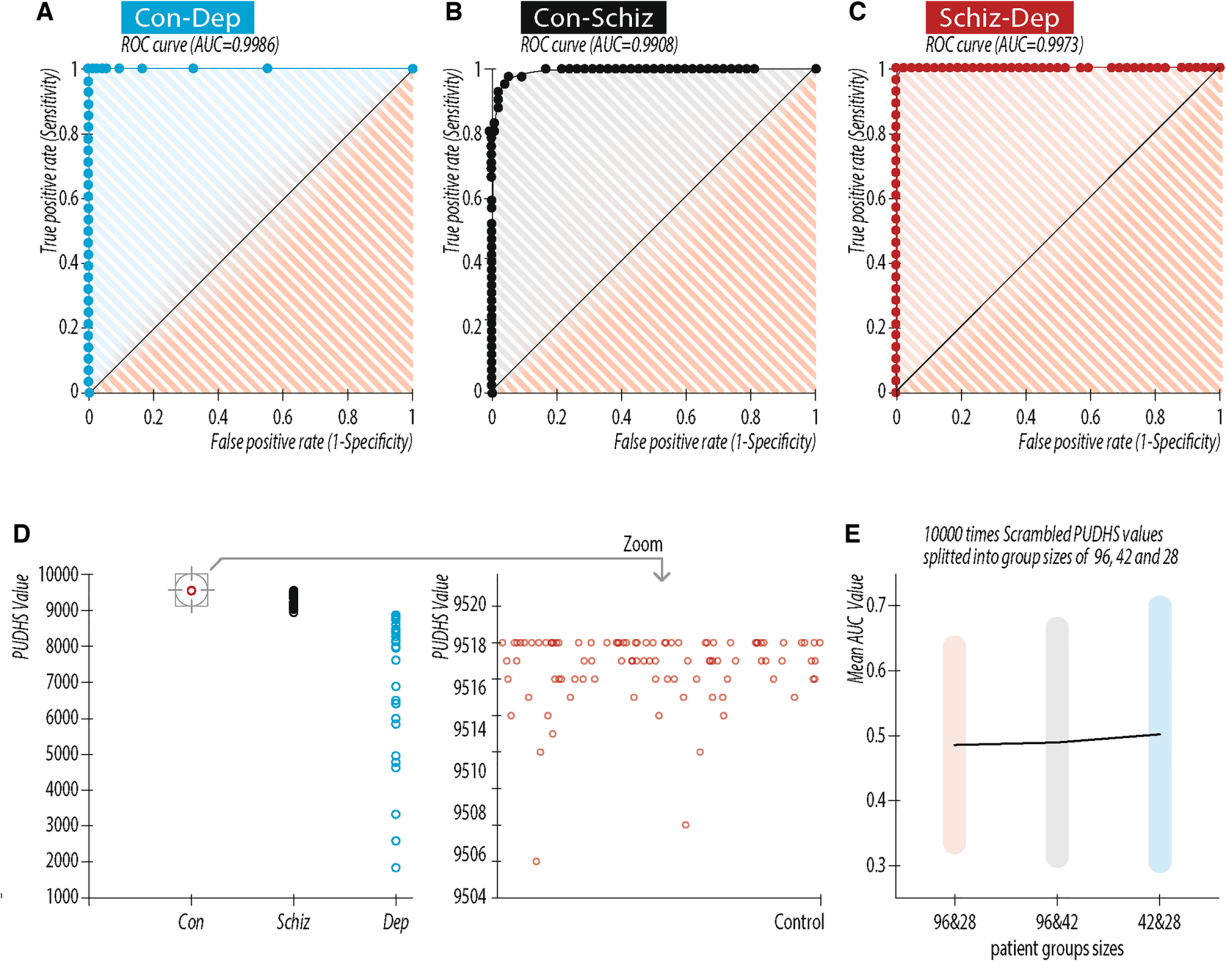
Accuracy of the EEG based personal universal DH signatures (PUDHSs) in differentiating control subjects from patients with schizophrenia and depression with artefact removal. Artefacts in the EEG caused by eye blinks, muscle activation, or other movement artefacts were removed for each patient’s EEG as described in the methods. Accuracy depicted as receiver operating characteristic (ROC) curves for (**A**) control vs. depression, AUC = 0.9986, p < 0.0001. (**B**) Control vs. schizophrenia 0.9908, p < 0.0001. (**C**) Schizophrenia vs. depression AUC = 0.9973, p < 0.0001. (**D**) Scatter plot of PUDHS values obtained from the control, schizophrenia, and depression patient groups. each range of PUDHS values belong exclusively to each clinical condition. each range of PUDHS values belong exclusively to each clinical condition. The insert shows a zoom-in of all control patients PUDHS values for better visuality. (**E**) Mean ± 3*std of AUC values obtained from ROC curves by 10,000 random grouping of patients into 3 groups with the size of 96, 42 and 28 respectively.

The topological structure of a patient’s dendrogram quantified as PUDHS shows high differentiation values for each patient group (all data following artefact removal; control: n = 96, 9516.6 ± 1.99; schizophrenia: n = 42; 9360.7 ± 1.62; p < 0.001; depression: n = 28; 6922.3 ± 2022.5; p < 0.001), Fig. 2D). The accuracy of the EEG-based PUDHSs in differentiating control subjects from patients with schizophrenia and depression is disclosed using receiver operating characteristic (ROC) curves. Control patients were differentiated highly accurately from patients with depression (AUC = 0.9986, p < 0.0001, Fig. 2A) and schizophrenia (AUC = 0.9908, p < 0.0001, Fig. 2B). Likewise, patients with schizophrenia showed high differentiation from those with depression (AUC = 0.9973, p < 0.0001, Fig. 2C). Artefacts in the EEG caused by eye blinks, muscle activation, or other movement artefacts were removed for each patient’s EEG as described in the methods.

To further ascertain that the patient groups themselves are distinct and to validate that the data does not contain other unwanted combinations of patients, we used 10 000 random groupings of all patients into three groups sized as our patient groups (96, 42 and 28) and calculated ROC of those 10 000 combinations (AUC: mean ± 3*STD, 96 & 28 = 0.485 ± 0.166; 96 & 42 = 0.489 ± 0.191; 42 & 28 = 0.501 ± 0.211; Fig. 2E). The separation capability of the PUDHS algorithm was kept robust and powerful even when artefacts were not removed (Fig S1). The AUC values are shown for control group vs schizophrenia group and schizophrenia group vs. depression group (control participants: n = 96, 9497.5 ± 2.1908; highly significantly different from participants with schizophrenia (n = 42; 8339.3 ± 966.6; p < 0.001) and depression (n = 28; 4139.1 ± 1867.7; p < 0.001), Fig. S1-D; control vs. depression: AUC = 0.9972, p < 0.0001 Fig. S1-A;control vs. schizophrenia: AUC = 0.9471, p < 0.0001, Fig. S1-B; schizophrenia vs. depression: AUC = 0.988, p < 0.0001, Fig. S1-C).

The impressive segregation between the three groups (patients with schizophrenia, depression, or control participants) is independent of the chosen EEG segment. We validated this by moving the 500 s period analyzed by 200 s. We thus verified that the differences in topology of the dendrograms, which represent relations between events, are stable even after changing the EEG segment of our analysis (Fig S2; EEG segment moved by 200 s; control: n = 96, 9516.3 ± 2.64; schizophrenia: n = 42; 8220.6 ± 1072.6; p < 0.001; depression: n = 28; 3622.9 ± 1823.4; p < 0.001, Fig. S2-D; control vs. depression: AUC = 0.9967, p < 0.0001, Fig. S2-A; control vs. schizophrenia: AUC = 0.9108, p < 0.0001, Fig. S2-B; schizophrenia vs. depression: AUC = 0.9863, p < 0.0001, Fig. S2-C). Changing the overall size of the dendrogram by using smaller dendrograms (fewer events) did not change the strength of segregation. Segregation values among the three groups were similar when the event analysis for each patient lasted for only 200 s of EEG recording (Fig S3; EEG segment lengths reduced to 200 s; control: n = 96, 3797.9 ± 1.80; schizophrenia: n = 42; 3144.7 ± 605.4; p < 0.001; depression: n = 28; 1553.5 ± 649.9; p < 0.001, Fig. S3-D; control vs. depression: AUC = 0.9972, p < 0.0001, Fig. S3-A; control vs. schizophrenia: AUC = 0.9457, p < 0.0001, Fig. S3-B; schizophrenia vs. depression: AUC = 0.9568, p < 0 0.0001, Fig. S3-C).

Our results indicate that EEG events show a specific relationship commonly found in controls participants but much less so in patients with schizophrenia and still less in patients with depression.

### Relationship-block subsystems

Clinical use of our method might be limited due to constraints of computational resources necessary for calculating dendrograms of the size described here. To tackle this, we dissected the EEG data as used above (500 s segments) into fragments of equal size (1 s, 3 s, 5 s, 10 s). For each sub-segment, we created a dendrogram accordingly. It is thus possible to construct a time series of smaller dendrograms, which significantly reduces the necessary computation power.

**Figure 3:**
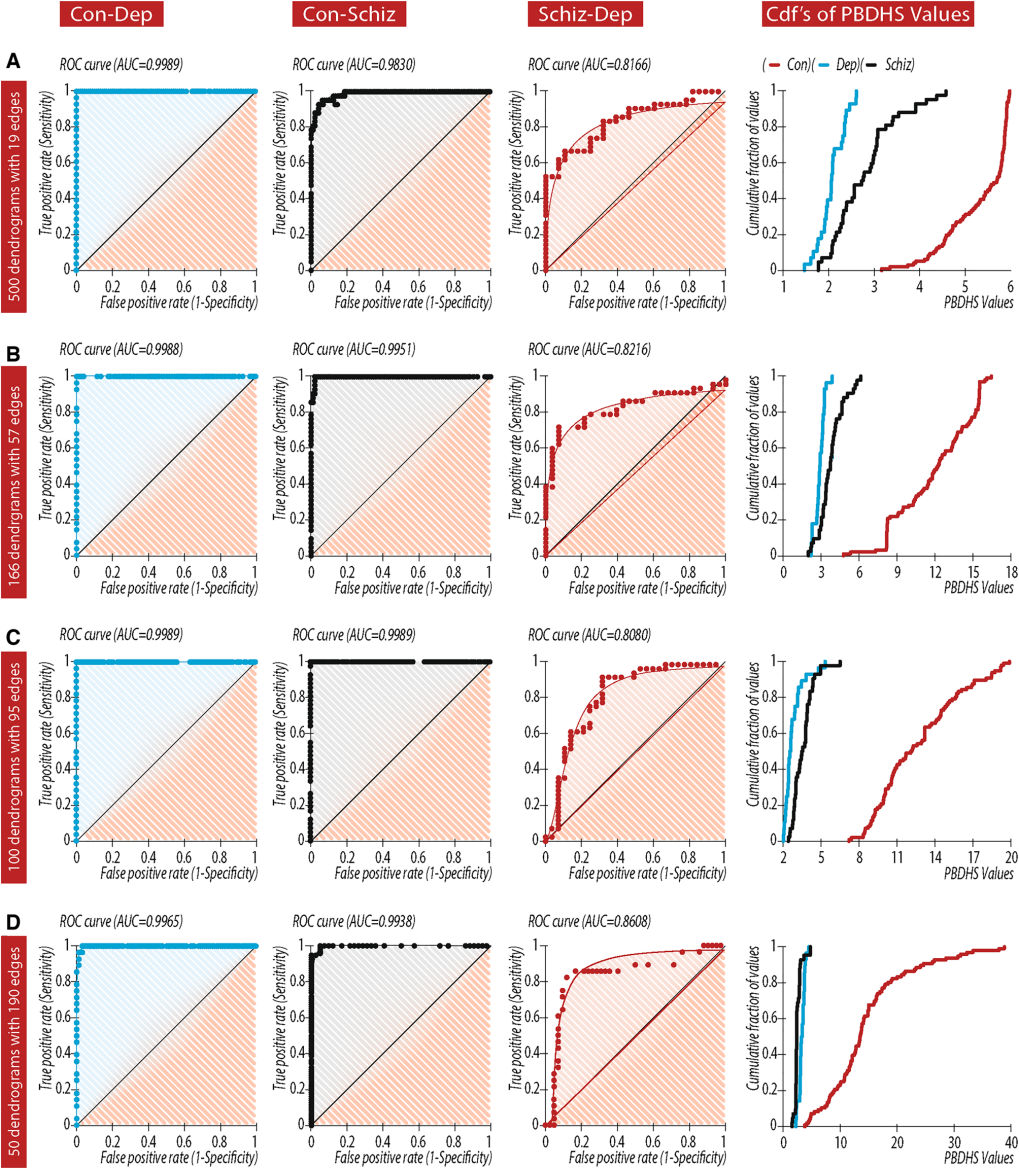
Accuracy of the EEG based personal block DH signatures (PUDHSs) in differentiating control, schizophrenia and depression patient groups. Accuracy depicted as receiver operating characteristic (ROC) curves for all pairs of clinical groups and CDFs of PBDHS values in each group. Each of the 500 s EEG recordings was separated into a time series of block dendrograms (**A**) 500 dendrogram of 19 edges each, (**B**) 166 dendrogram of 57, (**C**) 100 dendrograms of 95 edges. (**D**) 50 dendrograms of 190 edges.

The topological structures of each patient’s time series of dendrograms are characterized by PBDHS when using two thresholds, ${\mathrm{T}}_{\mathrm{dendrogram}}$ and ${\mathrm{T}1}_{\mathrm{dendrogram}}$, as described in the methods. Assuming that each group of patients is characterized by a different sub-system topological structure of dendrograms, the PBDHS will reflect these different topological structures as unique for each patient group. Choosing a dendrogram time series with different edges (19, 57, 95 or 190), we show a preserved discrimination power of those dendrograms for separating the patient groups (Fig. 3). Focusing on the dendrogram time series reflecting only spatial (and not temporal) features (19 simultaneous recordings from 19 electrodes and a second vector for EEG data), we obtained a high discrimination between patient groups (Table 1; Fig. 3A). By adding temporal features to the spatial separation of EEG data (57, 95 and 190 edges), separation between PBDHS values increased accordingly, which is reflected in the AUC values as well as in the PBDHS cumulative distribution function (CDF; Table 1, Fig. 3B–D). EEG artefact removal as described above as well as in the methods part did not change the segregation capability of the PBDHS (Fig. S4 and Table 1).

**Table 1:** Relationship-block subsystems PUHD values and ROC.

	PBDHS (mean ± SD)			AUC		
	Control	Depression	Schizophrenia	Control vs. depression	Control vs. schizophrenia	Depression vs. schizophrenia
1 s (19 edges)	5.2962 ± 0.6973	2.0735 ± 0.2864	2.8188 ± 0.6941	0.9989 p < 0.0001	0.9830 p < 0.0001	0.8166 p < 0.0001
3 s (57 edges)	12.2860 ± 2.6594	2.8663 ± 0.3934	3.7547 ± 0.9776	0.9988 p < 0.0001	0.9951 p < 0.0001	0.8216 p < 0.0001
5 s (95 edges)	12.4465 ± 3.2506	2.7894 ± 0.7948	3.5595 ± 0.8084	0.9989 p < 0.0001	0.9989 p < 0.0001	0.8080 p < 0.0001
10 s (190 edges)	14.7254 ± 7.3934	3.3087 ± 0.5599	2.6613 ± 0.7781	0.9965 p < 0.0001	0.9938 p < 0.0001	0.8608 p < 0.0001
10 s (190 edges) ICA artifacts rejection log2(BPDHS values)	16.2775 ± 5.0922	2.9302 ± 0.6714	3.8083 ± 0.8359	0.9981 p < 0.0001	0.9961 p < 0.0001	0.8446 p < 0.0001

PBDHS separation efficacy. PBDHS mean ± SD values for each patient group (first 3 columns) in each size of block dendrogram (rows 1–4). ROC analysis between pairwise patient groups (columns 4–6) in each size of block dendrogram (rows 1–4). Row 5 details analysis of EEG recording preprocessed with ICA artifact components rejection: first 3 columns-PBDHS mean ± SD values for each patient group in block dendrogram size of 190 events. columns 4–6 of Row 5 details ROC analysis between pairwise patient groups in block dendrogram size of 190 events.

### Topological and p-adic meanings of the threshold

**Figure 4:**
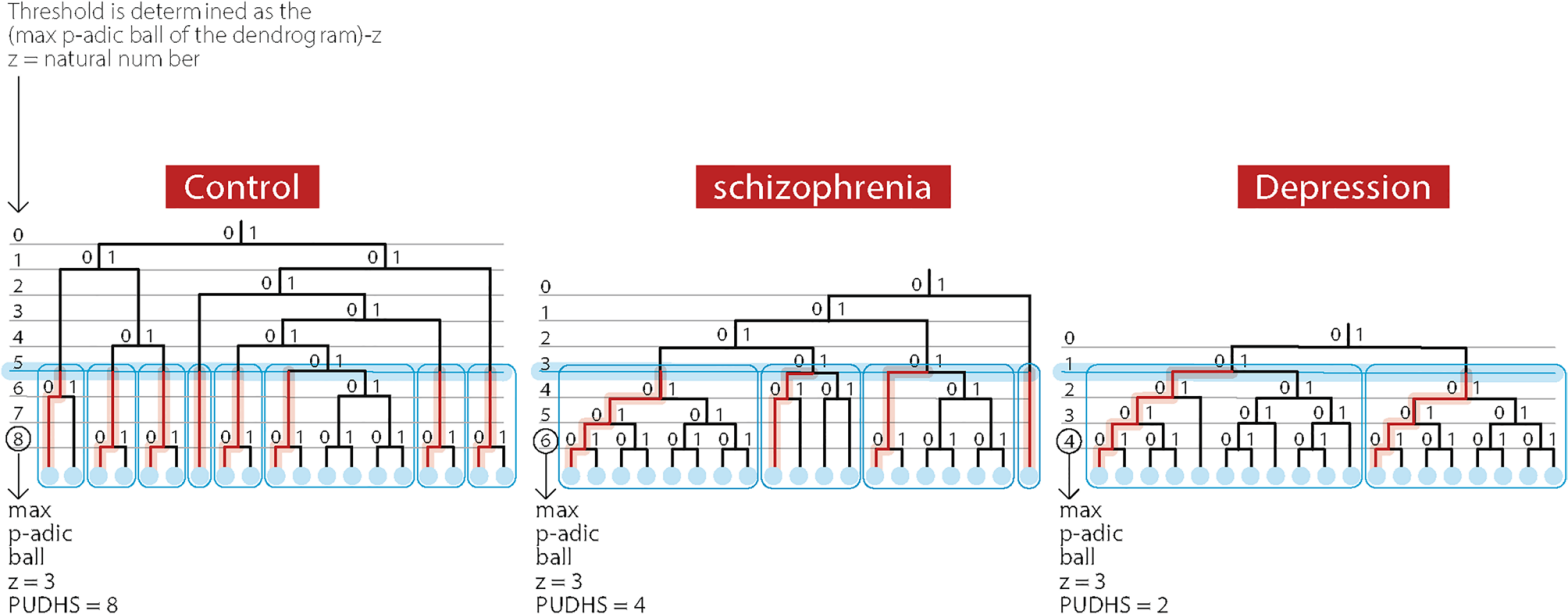
Illustration of dendrogramic topological differences between the control, schizophrenia, and depression groups. The max p-adic dendrogramic ball value is indicated by a circled level number on the left of each characteristic group dendrogram. The threshold, calculated as indicated (max p-adic ball of the dendrogram-Z), is seen as a thick blue horizontal line and creates disconnected subsystems (sub-dendrograms) of the main dendrogram. Each sub-dendrogram is encapsulated by a thin light blue square. The number of these sub-dendrograms, represent the PUDHS value.

The thresholds described have a simple topological and p-adical meaning. A threshold (graphically shown as a thick blue line in the characteristics dendrograms in Fig. 4) is defined as 2-adic ball value of each dendrogram disconnected subsystems (sub-dendrograms) of the main dendrogram (Fig. 4; thin light blue squares). These subsystems or sub-dendrograms merge at values that are lower than the threshold and closer to the root of the main dendrogram. Quantifying events that are lower than the threshold provides the number of disconnected subsystems with the same 2-adic ball radius. Thus, the PUDHS values indicate that in the control group, significantly more disconnected subsystems exist than e.g. in the schizophrenia group. Both control participants and patients with schizophrenia have more disconnected subsystems in their dendrograms than the group with depression. The distinct dendrogramic topology of each group of patients implies a simple physiological consequence: in the control group each EEG event is p-adically more distinct from each other, indicating that each event has its spatial location and temporal order, and serves as an autonomous information system that connects in a distinct manner to other spatio-temporal events. The information of events in the schizophrenia patients is comparably less segregated and more dispersed and even more so in the patients diagnosed with depression.

### EEG mean band power

Traditionally, EEG recordings are compared using regular band power methods. To compare our results with these classic analysis, we calculated for each band (alpha, beta, gamma, delta) the mean power of each electrode of each patient in the same 500 s data. We then calculated by ROC analysis the AUC values for each pair of patient groups at the same electrode (Fig. S5). AUC values of 0.7395 for comparing the 8^th^ electrode mean power values between control and depression patients were found. This value indicates a lower separation efficacy by regular power spectrum methods. Moreover, the mean powers of each bands in each patient group do not show significant differences (0.5–4 Hz: controls 60.2183 ± 190.7046, schizophrenia 46.0907 ± 62.4803, depression 99.0352 ± 355.9049; 4–8 Hz: controls 19.9440 ± 60.5356, schizophrenia 16.8882 ± 21.4367, depression 21.3134 ± 19.3526; 8–12 Hz, controls 18.6043 ± 20.6968, schizophrenia 15.6834 ± 12.4902, depression 19.8050 ± 16.0276; 12-30 Hz: controls 15.1538 ± 18.7423, schizophrenia 12.0568 ± 13.0867, depression 13.7774 ± 7.3449; 30–100 Hz: controls 30.8726 ± 52.5951, schizophrenia 74.3426 ± 159.5121, depression 36.9532 ± 82.5962).

## Discussion

The novel DH theory utilizes dendrogram representation of data as p-adic numbers to demonstrate the integration of the holistic brain function embodied in EEG signals with inert hierarchy of the brain signals. Namely, by extracting characteristic information patterns from dendrograms expressing the hierarchical treelike structure of information processing in the brain, encoded by p-adic numbers, we successfully differentiate and categorize the neuropsychiatric disorders depression and schizophrenia. Furthermore, AUCs showed high values, indicating high accuracy of differentiating patients with schizophrenia and depression from controls. This approach shows that the individual signatures described in the Results and Methods sections are useful for identifying those neuropsychiatric disorders.

Thus, it seems that during information processing, healthy brain functioning is characterized by significantly higher degrees of hierarchical interconnection, with high segregation of information across space and time compared to patients with schizophrenia or depression. The last statement, however, should be viewed with great caution, since EEG signals provide only a rough estimation of brain functioning.

The search for biomarkers that assist in diagnosing brain disorders has so far focused primarily on either biological samples, including serum or cerebrospinal fluid (CSF), or neuroimaging techniques, including magnetic resonance imaging (MRI) or functional MRI (fMRI). These methods are either invasive or expensive, and none has yielded an accurate biomarker for diagnosis of heterogeneous disorders such as major depression or schizophrenia^59–62^. Several EEG data analysis techniques have been used in recent studies in attempts to diagnose psychiatric disorders like schizophrenia^8–11^ and depression^12–16^. A machine-learning algorithm approach with promising results has been developed by Wu et al., who used resting-state EEGs to predict treatment responses in major depression^63^. Most studies use EEGs within a special research environment involving standardized situations, separate open and closed eye conditions, and artefact reduction. The clinical applicability of these methods for screening, diagnosis, and prediction of response to treatment is rather difficult and so we opted to use real-world EEG data. This enhances the generalizability but increases the risk that features of the clinical EEG recording environment might impact results. Recently, progress was made to accurately classify healthy subjects and patients with depression and schizophrenia by automated geometrical feature extraction of EEG signals^64–74^. The accuracy of these methods to separate these groups is non-inferior to our presented method, although their algorithm uses multiple feature extractions and thus the physiological interpretation is difficult. Contrary to this, we postulate an inherent distinction between information produced in the brain, both in a spatial and temporal fashion, in a disease dependent manner. We found a concrete and mathematically coherent method relying only on one feature namely the spatio-temporal connections of information signals of the brain.

It should be noted that the current authors initiated their study of hierarchical/p-adic representation of brain signals in a previous study that was recently published^49^. That study showed promising evidence of the power of hierarchical and topological features of dendrograms, quantified by the p-adic quantum potential, in discriminating among multiple neuropsychiatric disorders including depression, schizophrenia and cognitive decline. The method used previously relied on machine learning and quantified different topological features of dendrograms. A patient’s dendrogramic signature as described here provides a more refined characterization of mental state than of quantum potential. The relatively rough characterization of the relational hierarchy in information representation of the brain is a powerful enough mechanism for the quantification of cognition and diagnosis of mental disorders. Moreover, the new dendrogramic signature provides a unique range of values for each psychiatric disorder and mental state.

Further investigation is needed to study the combination of features quantified in the present study and expressed as patients’ dendrogramic signatures, alongside the quantum potential measure. Such comparative medical diagnostic studies may impact basic studies of cognition using PUDHS as a quantitative measure of cognitive processes. Moreover, further optimization of the threshold parameters is needed, especially considering the obvious expectation of applying this method to the diagnosis of other psychiatric, neurological and neurodegenerative disorders and to monitoring the response to treatment and progression of these disorders.

## Supplementary Information

Supplementary Figures.

## Data Availability

The datasets generated and/or analysed during the current study are available in the DRYAD repository, https://datadryad.org/stash/share/AWmC0-Afzx29cOkYDXQ6y2-7HF4GBvG-J-9i8hDQZsw.
