# Author Correction: EEG-based spatio-temporal relation signatures for the diagnosis of depression and schizophrenia

**DOI:** 10.1038/s41598-023-31604-w

**Published:** 2023-03-21

**Authors:** Oded Shor, Amit Yaniv-Rosenfeld, Avi Valevski, Abraham Weizman, Andrei Khrennikov, Felix Benninger

**Affiliations:** 1 Felsenstein Medical Research Centre, Petach Tikva, Israel; 2 Department of Neurology, Rabin Medical Centre, Petach Tikva, Israel; 3 Geha Mental Health Centre, Petach Tikva, Israel; 4 Faculty of Technology, Department of Mathematics, Linnaeus University, Vaxjö, Sweden; 5 Sackler Faculty of Medicine, Tel Aviv University, Tel Aviv, Israel; 6 Present Address: Shalvata Mental Health Centre, Hod Hasharon, Israel

Correction to: *Scientific Reports* https://doi.org/10.1038/s41598-023-28009-0, published online 14 January 2023

In the original version of this Article Andrei Khrennikov and Felix Benninger were omitted as equally contributing authors.

The original Article has been corrected.

