# Supplementary material for: EEG-based spatio-temporal relation signatures for the diagnosis of depression and schizophrenia
Source: Sci Rep. 2023 Jan 14;13:776. doi: 10.1038/s41598-023-28009-0 (PMC9840633; doi:10.1038/s41598-023-28009-0)
Supplement: Supplementary file 1 — Supplementary Figures. [file 41598_2023_28009_MOESM1_ESM.pdf]

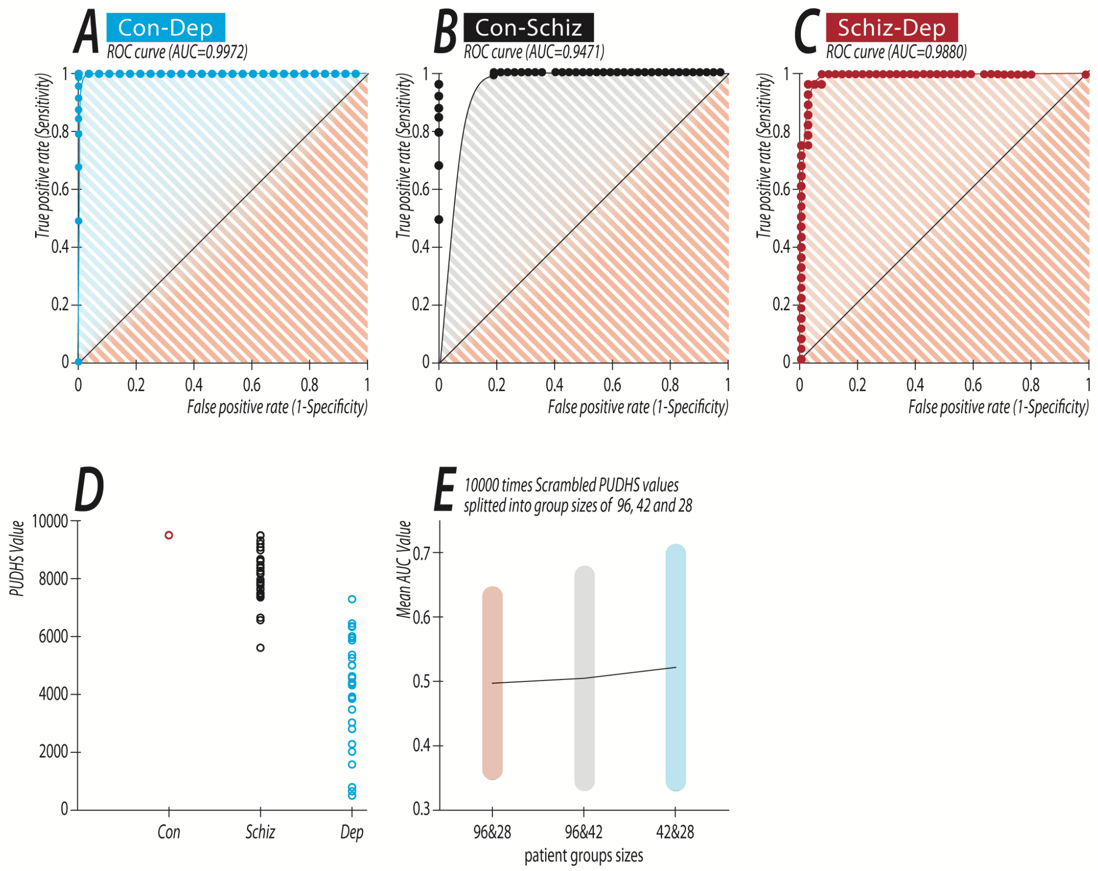

Supplementary Figure S1: Accuracy of the EEG based personal universal DH signatures (PUDHSs) in differentiating control subjects from patients with schizophrenia and depression without artefact removal. Accuracy depicted as receiver operating characteristic (ROC) curves for (A) control vs. depression, AUC=0.9972 p<0.0001. (B) control vs. schizophrenia AUC=0.9471 p<0.0001. C schizophrenia vs. depression AUC=0.988 p<0.0001. (D) Scatter plot of PUDHS values obtained from the control, schizophrenia, and depression patient groups. each range of PUDHS values belong exclusively to each clinical condition. each range of PUDHS values belong exclusively to each clinical condition. The insert shows a zoom-in of all control patients PUDHS values for better visuality. (E) mean±std of AUC values obtained from ROC curves by 10000 random grouping of patients into 3 groups with the size of 96,42 and 28 respectively.

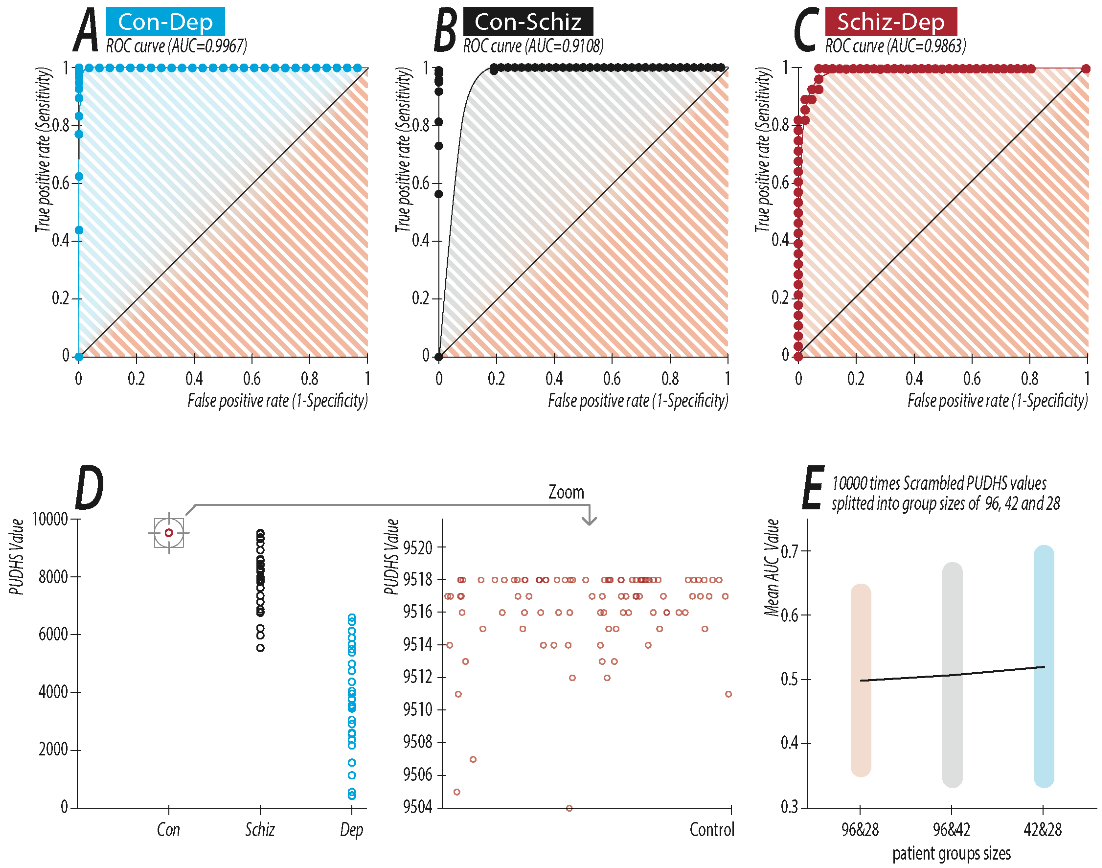

Supplementary Figure S2: Validation of the accuracy universal DH signatures (PUDHSs) by moving the EEG eindow (500s) by 200s. Accuracy depicted as receiver operating characteristic (ROC) curves for (A) control vs. depression, AUC=0.9967 $p<0.0001$. (B) control vs. schizophrenia AUC=0.9863 $p<0.0001$. C schizophrenia vs. depression AUC=0.9863 $p<0.0001$. (D) Scatter plot of PUDHS values obtained from the control, schizophrenia, and depression patient groups. each range of PUDHS values belong exclusively to each clinical condition. each range of PUDHS values belong exclusively to each clinical condition. The insert shows a zoom-in of all control patients PUDHS values for better visuality. (E) mean±3*std of AUC values obtained from ROC curves by 10000 random grouping of patients into 3 groups with the size of 96,42 and 28 respectively.

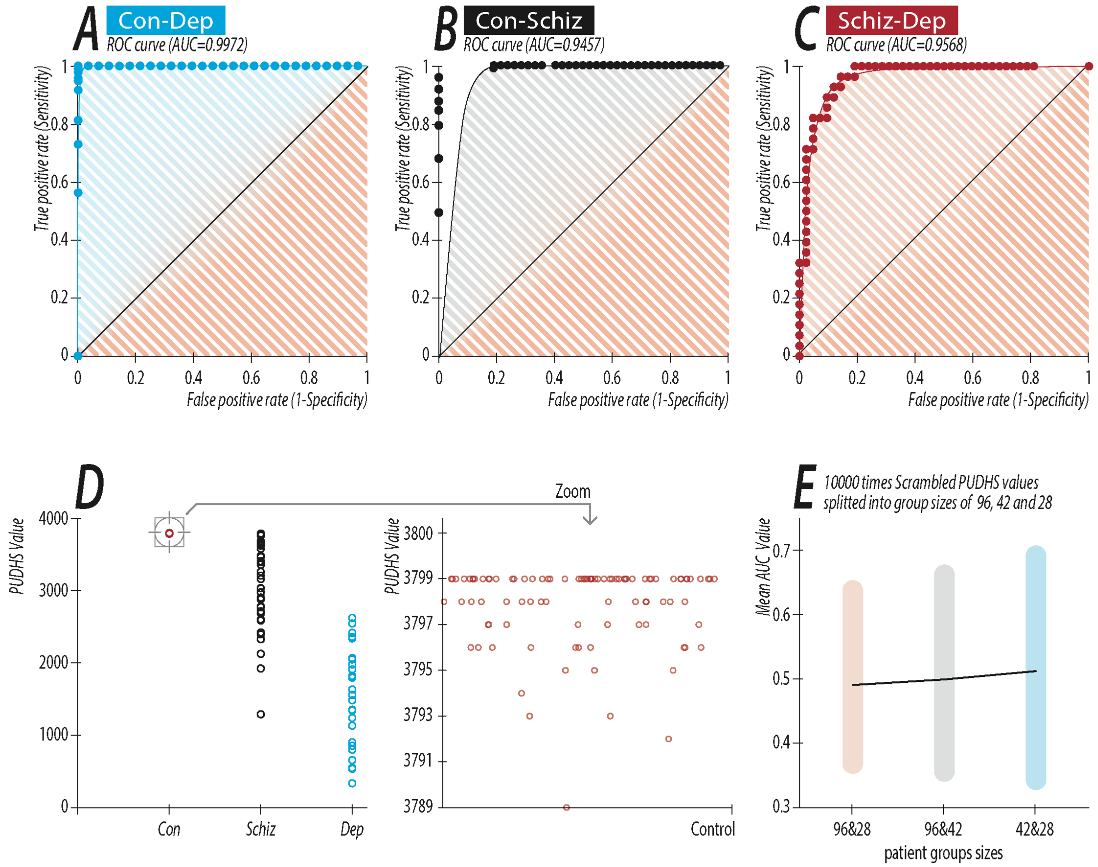

Supplementary Figure S3: Robustness of the accuracy of the universal DH signatures (PUDHSs) by shortening the EEG period tested to 200s. Accuracy depicted as receiver operating characteristic (ROC) curves for (A) control vs. depression, AUC=0.9972 p<0.0001. (B) control vs. schizophrenia AUC=0.9457 p<0.0001. C schizophrenia vs. depression AUC=0.9568 p<0.0001. (D) Scatter plot of PUDHS values obtained from the control, schizophrenia, and depression patient groups. each range of PUDHS values belong exclusively to each clinical condition. each range of PUDHS values belong exclusively to each clinical condition. The insert shows a zoom-in of all control patients PUDHS values for better visuality. (E) mean±3*std of AUC values obtained from ROC curves by 10000 random grouping of patients into 3 groups with the size of 96,42 and 28 respectively

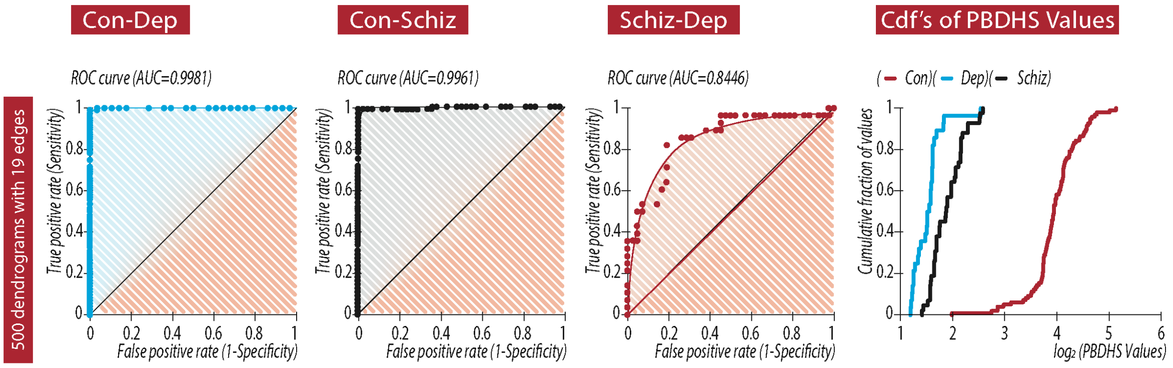

Supplementary Figure S4: EEG artefact removal as by ICA components removal of eye blinks, muscle activation, or other movement artefacts did not change the segregation capability of the PBDHS

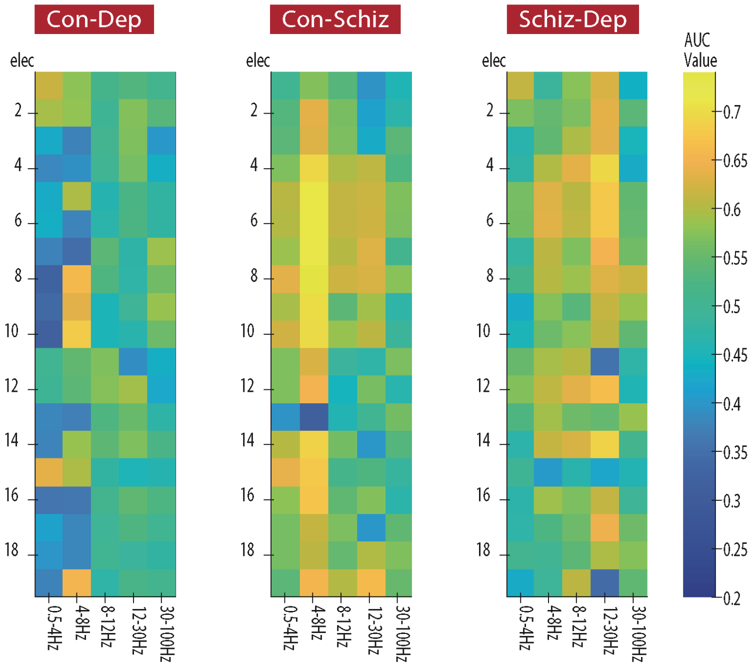

Supplementary Figure S5: Regular Band Power as complementary method to segregate patient groups. For each band (alpha, beta, gamma, delta) the mean power of each electrode of each patient in the same 500s data was calculated. AUC values (ROC analysis) for each pair of patient groups at the same electrode at each band are shown as a heat map.
